# Migratory chondroprogenitors retain superior intrinsic chondrogenic potential for regenerative cartilage repair as compared to human fibronectin derived chondroprogenitors

**DOI:** 10.1038/s41598-021-03082-5

**Published:** 2021-12-08

**Authors:** Elizabeth Vinod, Noel Naveen Johnson, Sanjay Kumar, Soosai Manickam Amirtham, Jithu Varghese James, Abel Livingston, Grace Rebekah, Alfred Job Daniel, Boopalan Ramasamy, Solomon Sathishkumar

**Affiliations:** 1 Department of Physiology, Christian Medical College, Vellore, India; 2 Centre for Stem Cell Research, (A Unit of InStem, Bengaluru), Christian Medical College, Vellore, India; 3 Department of Diabetes, School of Life Course Sciences, King’s College London, London, UK; 4 Department of Orthopaedics, Christian Medical College and Hospital, Vellore, India; 5 Department of Biostatistics, Christian Medical College, Vellore, India; 6 Department of Orthopaedics and Trauma, Royal Adelaide Hospital, Adelaide, Australia; 7 Faculty of Health and Medical Sciences, University of Adelaide, Adelaide, Australia

**Keywords:** Cell biology, Stem cells

## Abstract

Cell-based therapy for articular hyaline cartilage regeneration predominantly involves the use of mesenchymal stem cells and chondrocytes. However, the regenerated repair tissue is suboptimal due to the formation of mixed hyaline and fibrocartilage, resulting in inferior long-term functional outcomes. Current preclinical research points towards the potential use of cartilage-derived chondroprogenitors as a viable option for cartilage healing. Fibronectin adhesion assay-derived chondroprogenitors (FAA-CP) and migratory chondroprogenitors (MCP) exhibit features suitable for neocartilage formation but are isolated using distinct protocols. In order to assess superiority between the two cell groups, this study was the first attempt to compare human FAA-CPs with MCPs in normoxic and hypoxic culture conditions, investigating their growth characteristics, surface marker profile and trilineage potency. Their chondrogenic potential was assessed using mRNA expression for markers of chondrogenesis and hypertrophy, glycosaminoglycan content (GAG), and histological staining. MCPs displayed lower levels of hypertrophy markers (RUNX2 and COL1A1), with normoxia-MCP exhibiting significantly higher levels of chondrogenic markers (Aggrecan and COL2A1/COL1A1 ratio), thus showing superior potential towards cartilage repair. Upon chondrogenic induction, normoxia-MCPs also showed significantly higher levels of GAG/DNA with stronger staining. Focused research using MCPs is required as they can be suitable contenders for the generation of hyaline-like repair tissue.

## Introduction

Articular cartilage, a specialized tissue, plays a vital role in ensuring frictionless movement between the articulating bone surfaces^1^. Cartilage loss following trauma, disease, or age-related wear and tear often progress to arthritis, eventually necessitating joint replacement. Current pharmacological and surgical therapies enable attenuation of symptoms but fail to provide a long-standing solution towards the restoration of hyaline articular cartilage. In recent years, cartilage repair using cellular therapeutics for regeneration of hyaline-like cartilage tissue has gained prominence. The commonly employed cells are autologous chondrocytes and mesenchymal stem cells (MSCs)^2^. Even though various reports show their therapeutic efficacy, the major limitation is that the generation of repair tissue consists of mixed hyaline/fibrocartilage, which results in inferior biomechanical and functional outcomes^3–6^.

Circumstantial evidence supporting the presence of precursors, and studies to understand their role in appositional growth led to the discovery and isolation of cartilage resident progenitors^7–9^. The potential of articular cartilage-derived chondroprogenitors has recently gained interest due to their phenotypic predisposition for chondrogenesis and reduced hypertrophic proclivity. As compared to chondrocytes and bone marrow-derived MSCs, chondroprogenitors demonstrate supremacy in terms of higher chondrogenic potential and lower expression of fibrocartilage/hypertrophy markers such as Collagen type I and RUNX2^10–13^. As a result, these cells open up new possibilities and strategies for cartilage regeneration and tissue engineering.

Isolation of these progenitors commonly employs loading of chondrocytes on fibronectin adhesion assay^14,15^ or sorting based on putative surface markers^16,17^. Another standard method to obtain progenitors is based on their migratory potential from cartilage explants in culture^8,18^. Both fibronectin adhesion assay derived^19,20^ and migratory chondroprogenitors^8,21^ have been likened to MSCs as delineated by the International Society for Cellular Therapy (ISCT) 2006^22^, demonstrating plastic adherence, similar surface marker expression, and trilineage differentiation potential. Extensive work on these progenitors has provided information on their growth characteristics, where fibronectin-derived cells displayed clonal growth and required additional growth factors for expansion, unlike migratory progenitors^23^. As compared to bone marrow-MSCs, independent in- vivo animal studies using these progenitors show that they are more effective in repairing chondral defects^11,24^. Despite the fact that both types of progenitor cells, though isolated using different protocols, have features that are suitable for neocartilage formation, a direct comparison of the two cell groups may aid in determining which one demonstrates the better potential for cartilage tissue engineering.

Chondrogenesis involves a complex multiparametric differentiation process that is influenced by the cellular microenvironment. Multiple reports show that successful chondrogenic differentiation of MSCs depends on the three-dimensional culture environment^25^, a cocktail of chondrogenic growth factors^26^, and hypoxia^27^. In an in-vitro setting, standard culture conditions use 21% oxygen (O_2_), which is hyperoxic when compared to the in-vivo cartilage microenvironment. Oxygen tension within the cartilage, proximal to the subchondral bone, is estimated to be as low as 1%^28^. Consequently, investigations on the effects of hypoxia on chondrocytes have shown to promote chondrogenesis, redifferentiation, and hinder the expression of hypertrophic markers in both non-diseased and osteoarthritic cells^29–31^. Though there have been a few studies demonstrating a beneficial outcome of hypoxia in terms of mechanical properties, collagen content, and downregulation of hypertrophy genes on fibronectin assay-derived chondroprogenitors^32,33^ relative to normoxia culture conditions, to our knowledge, no studies have reported the effects of hypoxia on migratory chondroprogenitor chondrogenesis.

This study aimed to compare the chondrogenic potential of fibronectin adhesion assay and migratory progenitors isolated from human osteoarthritic knee joints, to evaluate differences in their biological characteristics and potential for cartilage repair. In addition, the study also investigated whether chondroprogenitors, grown in sustained hypoxic conditions (mimicking the natural environment), could display enhanced intrinsic chondrogenic potential.

## Methods

### Study design

All experimental protocols were approved by the Institutional Review Board (Research and Ethics Committee) of the Christian Medical College, Vellore. All methods were carried out in accordance with relevant guidelines and regulations.

**Figure 1:**
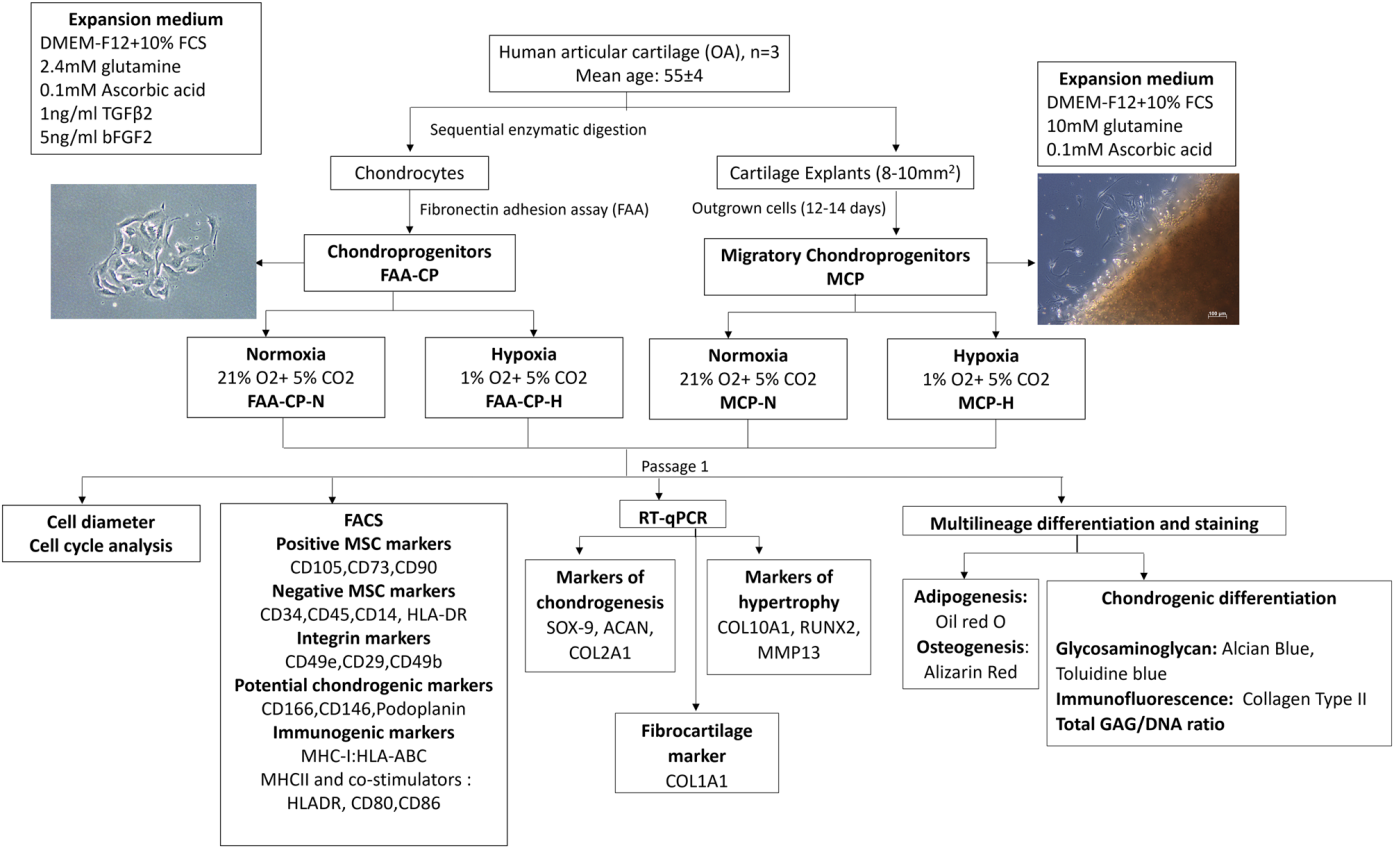
Study algorithm depicting the four groups used for comparison and their evaluation parameters. The four groups were fibronectin adhesion assay-derived chondroprogenitors cultured under normoxia (FAA-CP-N) and hypoxia (FAA-CP-H), migratory chondroprogenitors cultured under normoxia (MCP-N), and hypoxia (MCP-H). OA: osteoarthritis, CD: cluster of differentiation, SOX-9:(sex-determining region Y)-box 9, ACAN: aggrecan, COL: collagen, RUNX2: Runt-related transcription factor-2, MMP-13: matrix metalloproteinase-13, GAG: glycosaminoglycans. The experiments were conducted with three biological samples (n = 3).

After obtaining written informed consent from all subjects, human cartilage samples were harvested from three osteoarthritic (OA) knee joints of patients [age (mean ± SD): 55 ± 4 years], undergoing total knee replacement. These patients had high-grade osteoarthritis (Kellgren-Lawrence radiological score of 4). Patients whose knee joints had tumors, inflammation, or infection were excluded from the study. The cartilage slices were subjected to two different methods of isolation to obtain the individual chondroprogenitor subsets. Following enzymatic digestion, the released chondrocytes were subjected to selective adhesion assay using fibronectin to obtain progenitors. These subsets will hereon be referred to as fibronectin adhesion assay-derived chondroprogenitors (FAA-CP). Standard explant cultures were performed using cartilage slices taken from the surface of the knee joints. After 12 days of culture, outgrown cells were harvested and further cultured to obtain migratory chondroprogenitors (MCP). Both FAA-CP and MCP were expanded either under normoxia (21% O_2_ and 5% CO_2_) and hypoxia (1% O_2_ and 5% CO_2_). This study involved four groups for comparison, where FAA-CP and MCP were maintained in either normoxia or hypoxia culture conditions from their time of isolation to expansion and evaluation. The cells were expanded to passage 1 and subjected to characterization using fluorescence-activated cell sorting (FACS) for surface marker expression, studies using cell cycle analysis, quantitative reverse transcriptase-polymerase chain reaction (qRT-PCR) for markers of chondrogenesis and hypertrophy, biochemical analysis of chondrogenic differentiated pellets for total GAG (glycosaminoglycan)/DNA content, and trilineage differentiation with confirmatory staining (Fig. 1). The experiments were conducted with three biological samples (n = 3).

### Isolation and culture of FAA-CP

Cartilage slices from OA knee joints were washed and minced to a size of 1–2 mm. The FAA-CPs were isolated as per protocol previously described by Nelson et al.^15^ In brief, the minced cartilage was subjected to sequential cellular digestion using pronase (12 IU, Roche) for 3 h followed by overnight incubation with collagenase type II (100 IU, Worthington) at 37 °C. The released cells were loaded on fibronectin-coated plates (10 µg/ml, Sigma) at a concentration of 4000 cells per well for 20 min. Following this, the non-adherent cells and medium were removed and replaced with stromal medium containing DMEM/F12 (Sigma), 10% fetal bovine serum (FBS, GIBCO, Thermo Fischer Scientific), ascorbic acid (0.1 mM, Sigma), L-glutamine (2.4 mM/L, Sigma), antibiotics, and antimycotics. The adherent cells were maintained as previously described in either normoxic or hypoxic culture conditions for 12 days to obtain clones of > 32 cells (minimum population doubling of 5). The clones were isolated and replated at a ratio of 1 clone/cm^2^. Further expansion of enriched polyclonal chondroprogenitor cultures to passage 1 was done as per previously reported protocol^15^. The cells were expanded in a stromal medium containing human recombinant transforming growth factor beta2 (TGFβ2, Biovision) at 1 ng/ml, and human recombinant fibroblastic growth factor (FGF2, Biovision) at 5 ng/ml, as per standard established protocol.

### Isolation and culture of migratory chondroprogenitors (MCP)

**Figure 2:**
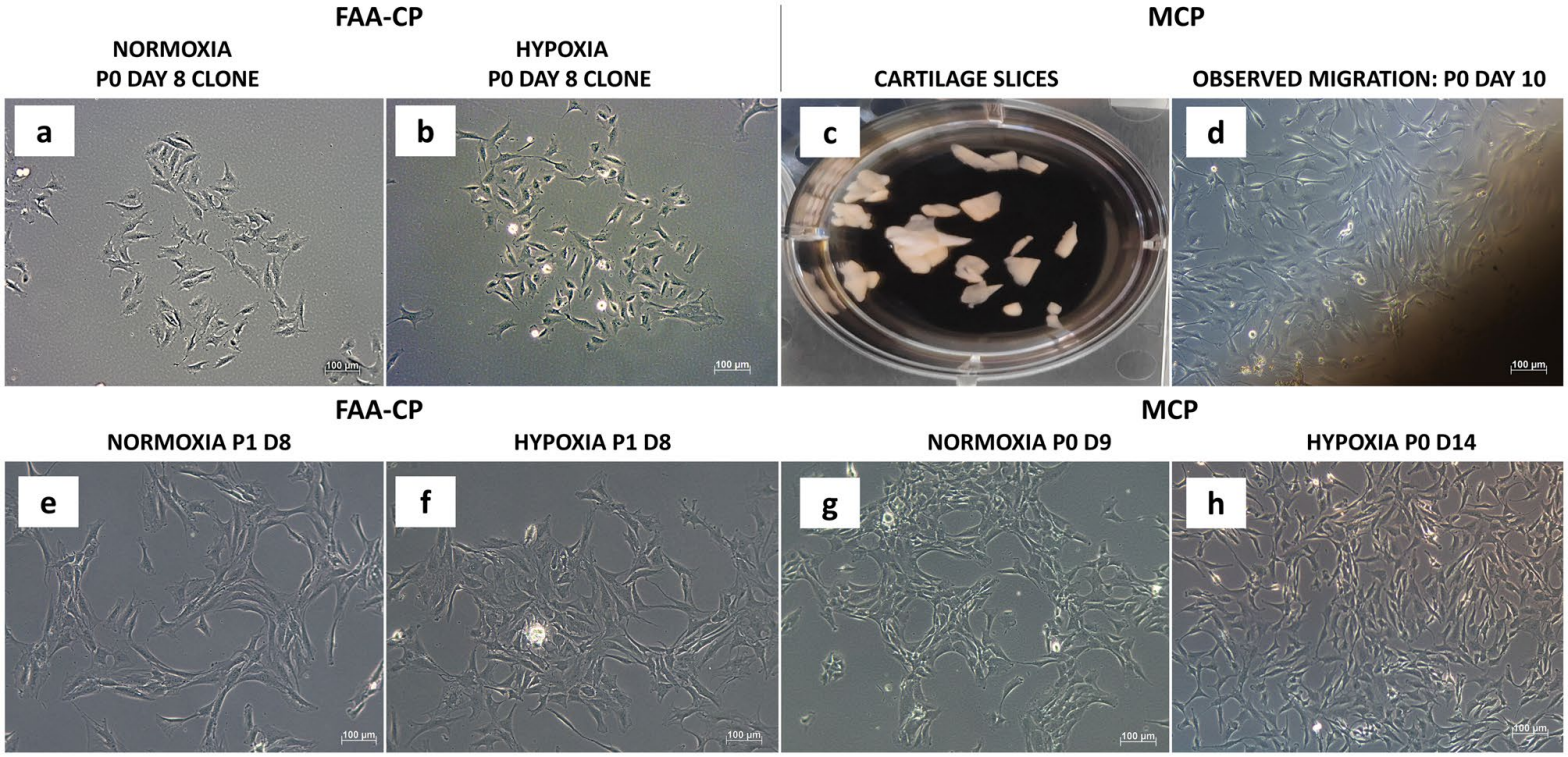
Representative images of FAA-CPs and MCPs during monolayer expansion culture under normoxic and hypoxic conditions. Microscopic analysis observed using phase-contrast microscopy showed that both normoxia and hypoxia FAA-CPs (**a**,**b**, passage 0) demonstrated clonal growth. Migration of chondroprogenitors from the cartilage explants was seen by the 7th day (**c**,**d**, passage 0) in both the microenvironments. Monolayer expanded passage 0 cells in all four groups displayed a comparable spindle-shaped morphology with a typical honeycomb pattern of growth, Magnification 10x (**e**–**h**).

Cartilage slices of 8–10 mm were harvested and placed in DMEM/F12 containing 10% FBS, 0.1 mM ascorbic acid, 10 mM Glutamine, antibiotics, and antimycotics (Fig. 2). Isolation was performed as per the protocol described by Koelling et al. and Wang et al^8,24^. In brief, following equilibration for a minimum period of 48 h the cartilage explants were placed in 0.1% collagenase type II (Worthington) for a period of 2 h at 37 °C. Further, the cartilage slices, devoid of any released cells were washed, and placed in a culture plate containing to stromal medium in a culture plate and observed for the outgrowth of cells. After 10 days, the migrated cells were harvested and further expanded to passage 1 for further characterization.

For all cultures, the medium was changed once every three days, and at sub-confluence (85–90%), cell harvest was carried out using 0.125% Trypsin-EDTA (GIBCO, Thermo Fischer Scientific). It was ensured that the culture, expansion, and differentiation of the four groups (FAA-CP vs MCP, normoxia vs hypoxia) were performed under their specified culture conditions. Since the standardized culture conditions recommend the requirement of additional growth factors for FAA-CPs^14,15,32^ but not for MCPs^8,34,35^, the two types of chondroprogenitors were cultured accordingly.

### Cell diameter and cell cycle analysis

The percentage of cells in different phases of the cell cycle was analyzed using DAPI (4’,6-diamidino-2-phenylindole). At a confluence of 60–70% and a day after medium change, the cells were trypsinized. Cell size estimation was done using an automated counter (Countess, Invitrogen). Passage 1 chondroprogenitors at a concentration of 1 × 10^6^ cells were washed with phosphate buffer solution (PBS) and fixed with cold 70% ethanol for 1 h. Following a wash, DAPI at a concentration of 1 µg/ml (in PBS containing 0.1% TritonX100) was used for incubation for a duration of 30 min. The washed cells were resuspended in PBS and subjected to flow analysis. The Dean Jett algorithm was used to analyze the percentage of cells in various phases using Flo-Jo software. The experiments were conducted with three biological samples (n = 3).

### Surface marker expression using FACS

At sub confluence (< 90%), passage 1 chondroprogenitors (biological replicates, n = 3) were trypsinized, washed with PBS and processed for FACS. The staining method followed was as per the instruction manual provided with the individual antibodies. Since chondroprogenitors have been likened to MSCs, the first group of surface markers considered for comparison included markers of positive expression: CD105-FITC, CD73-PE, CD90-PE, and markers of negative expression: CD34-APC, CD45-FITC, and CD14-FITC. The second group for comparison included integrin markers: CD 49e-PE, CD29-APC, and CD49b-FITC. The third group for comparison included CD markers which are reported to be potential markers of enhanced chondrogenesis: CD166-BB515^13,36^, CD146-PE^17,37^, and Podoplanin-BV421^38^. The final group included human leucocyte antigen class I (HLA-ABC-PE), HLA class II (HLA-DR V500), and their co-stimulatory molecules, CD80-BB515 and CD86-BV421. In brief, cells were washed in PBS and stained according to the manufacturer's instructions for individual antibodies (Supplementary Table S1). Data acquisition was done using BD FACS Cytoflex cytexpert. Unstained controls were run in parallel, and data were analyzed using BD FACS Diva v 8.0.1.1 and Flo Jo software.

### Quantitative reverse transcriptase-polymerase chain reaction (qRT-PCR)

The harvested cells were washed in PBS and subjected for total RNA extraction using Qiagen RNeasy Mini Kit as per the manufacturer’s instruction. The absorbance at 260 nm and 280 nm (A260/A280) was used to determine the RNA concentration using a nanodrop spectrophotometer. Complementary DNA was synthesized with 280 ng of RNA, using the Takara Bio First-Strand synthesis system. PCR assays were carried out using Takyon Low Rox SYBR Master Mix Dttp Blue (Eurogentec, Belgium) on a QuantStudio 6 K Flex thermocycler (Applied Biosystem). The expression of chondrogenic marker genes (SOX-9, ACAN, and COL2A1), fibrocartilage marker gene COL1A1, and hypertrophic chondrocyte marker genes (COL10A1, RUNX2, and MMP-13) was measured. The relative mRNA expression for all target genes was normalized to glyceraldehyde 3-phosphate dehydrogenase (GAPDH) to obtain the ΔCt. GAPDH was utilized as the housekeeping gene as it demonstrated high and stable expression under varying conditions (data not shown). The obtained ΔCt values were compared to the normoxia FAA-CP group (ΔΔCt), from which 2^− ΔΔCt was calculated. In addition, the ratio of COL2A1 to COL1A1 was calculated to evaluate their hyaline cartilage forming potential. The experiments were conducted with three biological samples (n = 3) in two technical replicates. Specific primer sequence (5’-3’ and 3’-5’), accession code, gene identifier, and base-pair length for the eight genes are listed in Supplementary Table S2.

### Multilineage differentiation and confirmatory staining

Induction towards adipogenic, osteogenic, and chondrogenic lineage was performed using StemPro differentiation kits (Thermo Fischer, Cat no: A1007201, A1007001, and A1007101). Before adipogenic and osteogenic differentiation, cells were seeded at a concentration of 5000 cells/cm^2^ in 24 well culture dishes and expanded to sub confluence. Then, the stromal media was replaced with the differentiation media. The control arm included cells grown in a stromal medium for the same period. For three-dimensional pellet cultures, 0.5 × 10^6^ cells were loaded into Eppendorfs, centrifuged at 400 g for 12 min, and left uninterrupted for 24 h. Following this, StemPro chondrogenic medium was added to the pellets. The medium was changed every three days for a period of three weeks.

### Adipogenic and osteogenic staining

Differentiated adipocytes were fixed for 1 h with 10% formaldehyde, washed, and stained with Oil Red O (Sigma), while differentiated osteocytes were fixed for 1 h with 70% ethanol, washed, and stained with Alizarin Red (Sigma). Parallel controls were also subjected to staining and imaging using the Olympus virtual slide system.

### Chondrogenic staining

Following differentiation, the pellets were washed and fixed with 4% paraformaldehyde for a period of 15 min. The cassettes containing the pellets were dehydrated for 10 min in a series of ethanol solutions of increasing concentration (70 to 100%). This was followed by ethanol clearance using xylene, wax infiltration, and paraffin embedding into mold cassettes. Paraffin sections (4 µm) were obtained using a microtome on poly-L lysine (PLL) coated slides and kept at 65 °C for 2 h. The sections were hydrated with descending grades of alcohol (100% to 70%) and washed prior to processing. For confirmation of differentiation the PLL sections were washed and stained with Alcian Blue dye, pH:2.5 (Cat no: J60122, Alfa Aesar, US) for 15 min, and counterstained using neutral red for 1 min. For Toluidine blue staining, the sections were stained with 0.1% Toluidine blue (C.I No-52040 Qualigens) solution for 5 min, washed, and dipped in 95% alcohol. For Picrosirius red staining, the sections were treated with 0.1% Picrosirius red (C.I.35782) for 1 h and counterstained with Hematoxylin for 7 min. Following staining, all slides were dehydrated with a series of graded alcohol and cleared in xylene before mounting with glycerol. Cell pellets cultured with stromal expansion medium, not subjected to chondrogenic differentiation served as controls (Supplementary Fig. S1).

### Immunofluorescence (IF) staining: Collagen type II

Paraffin slides were kept at 65 °C for 2 h. Following treatment with xylene, the pellets were hydrated with descending grades of alcohol, washed, and treated with 0.1% PBST (0.1% TritonX100). Antigen binding sites were retrieved using 1 mg/mL pronase and 5 mg/ml hyaluronidase incubation at 37 °C for 30 min each. The slides were subjected to protein block [1% bovine serum albumin (BSA) and 6% FCS] for 30 min, following which primary mouse monoclonal Anti-Collagen type II antibody [DSHB Hybridoma Product II-II6B3] was added at a concentration of 5 µg/mL and incubated overnight at 4 °C. Following the protein block, the sections were incubated with 1:100 secondary antibody [IgG (H + L) highly cross-adsorbed Goat anti-Mouse, Alexa Fluor 594, Invitrogen (Catalogue no: A11032)] for 30 min. Counterstaining was performed with 10 µg/ml DAPI (Sigma) for 5 min and mounted with 90% glycerol.

The images of Alizarin red S and Oil red O were acquired using Leica DMIL microscope, routine histological stains with Olympus bx43f. microscope, and Collagen type II with Olympus fv1000 laser scanning confocal microscope.

### Biochemical analysis: total GAG/DNA content of chondrogenic pellets

For quantification of GAGs and DNA, pellets were collected 3 weeks after chondrogenic induction and digested with 120 µg/ml papain at 65 °C for 16 h. DNA content was measured by Quant-iT Picogreen dsDNA reagent, and Lambda DNA was used for the generation of the standard curve. Fluorescence intensity was measured using the SpectraMax i3x Reader (Norwalk, CT, USA) at an excitation wavelength of 480 nm and an emission wavelength of 520 nm. GAG content was then quantified using the dimethyl methylene blue dye method according to the manufacturer’s instructions (Chondrex, Cat No: 6022). Chondroitin 6-sulfate was used as a standard. The optical density was measured at 525 nm and total GAG content was calculated. For comparison of GAG production, GAG content was normalized to DNA content (GAG/DNA).

### Statistical analysis

Data analysis was performed using SPSS version 21.0 (SPSS Inc, Chicago, USA) and graphical presentation using Prism v6 (GraphPad). The One-way ANOVA with post hoc LSD correction was used to assess the significance of differences between the four groups. Numerical values were expressed as mean ± standard error mean. A *P* value of < 0.05 was considered to be significant.

## Results

### Cell diameter and cell cycle analysis

**Figure 3:**
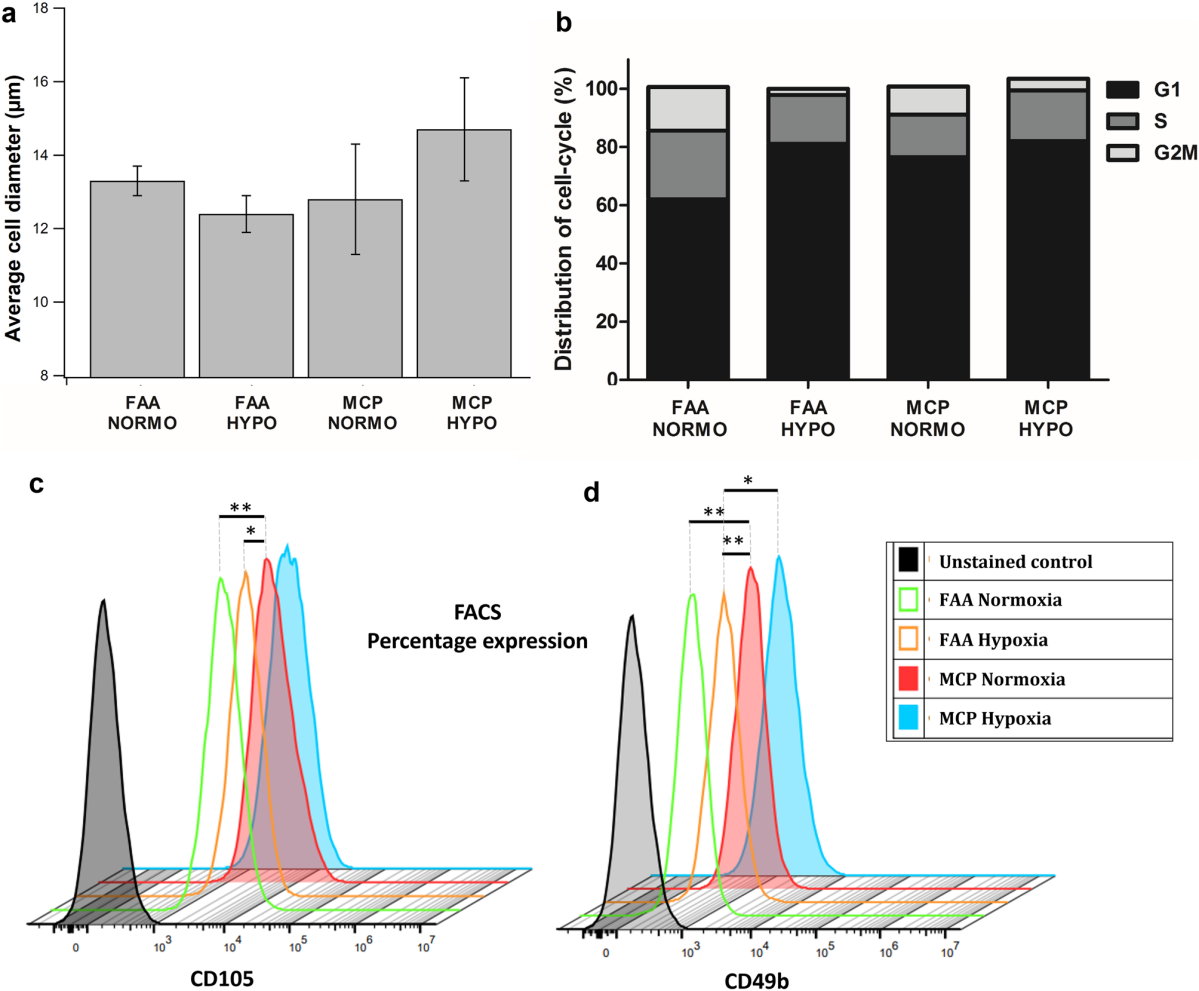
(**a**) Analysis of the average cell diameter (µm) of passage 1 chondroprogenitors showed that hypoxia MCPs and normoxia FAA-CPs displayed greater diameter than their counterparts although not statistically significant. (**b**) Analysis of the different phases in cell cycle distribution between the groups showed that both FAA-CPs and MCPs grown under hypoxia displayed a greater percentage at G1 interphase and lower percentage at G2M interphase as compared to their normoxic counterparts. However, when the DNA synthetic S phase was compared the highest values were observed with normoxia FAA-CPs. (**c**) To characterize the surface marker expression profile, FAA-CPs and MCPs cultured under normoxia and hypoxia were subjected to fluorescence-activated cell sorting. All groups revealed comparable expression of CD markers (Table 1) except for CD105 and CD49b (**c** and **d**: staggered plot). MCP-Normoxia expressed significantly higher levels of both CD105 and CD49b when compared to both FAA-CP-Normoxia and FAA-CP-Hypoxia (*P* < 0.01). In addition, MCP-Hypoxia also displayed a higher expression of the integrin marker CD49b when compared to the FAA-CP-Hypoxia cell group. Data presented are expressed as Mean ± standard error mean. All experiments were conducted with three biological samples (n = 3).

Both normoxia and hypoxia FAA-CPs demonstrated clonal growth, achieving a population doubling of 5 by day 12 (Fig. 2a,b). Concerning MCPs, migration of chondroprogenitors from cartilage explants was observed as early as day 7 (Fig. 2d). On further expansion, both the cell groups displayed spindle-shaped morphology with a honeycomb pattern of growth, a hallmark of the MSC population (Fig. 2e–h). When the average cell diameter (µm) of passage 1 cells (n = 3) were compared, there was no significant difference observed between the groups (Fig. 3a). Analysis of the different phases in cell cycle distribution between the groups showed that both FAA-CPs and MCPs expanded under hypoxia displayed a greater percentage at G1 interphase and lower percentage at G2M interphase as compared to their normoxic counterparts. However, when the DNA synthetic S phase was compared the highest values were observed with normoxia FAA-CPs. The observed differences were not significant, though the MCPs were expanded in a stromal medium devoid of additional growth factors (Fig. 3b).

### Surface marker expression using FACS

Flow cytometric analysis of the two populations was performed to study surface marker expression based on the mentioned categories. When MSC markers were compared, all the groups (n = 3) displayed a high expression of positive markers CD105, CD73, and CD90, and a low expression of negative markers CD34, CD45, and CD14 (Table 1). There was no significant difference between the groups except for CD105, which was higher in normoxia MCP when compared to both normoxia and hypoxia FAA-CP (*P* < 0.01, Fig. 3c). Regarding the integrin markers, all the groups showed a high expression of CD49e and CD29, with no significant difference, except for CD49b, which was higher in the normoxia MCP group as compared to FAA-CPs (*P* < 0.05, Fig. 3d). The hypoxia MCP showed higher levels of CD49b when compared to the hypoxia FAA-CP group alone (*P* = 0.036). When potential markers of chondrogenesis were assessed, all the groups demonstrated high and comparable levels of expression (Table 1). Concerning the immunogenic markers, all the groups displayed a comparable and positive expression of HLA-ABC, with a low expression of MHC II and its co-stimulatory molecules.

**Table 1:** Fluorescence-activated cell sorting data for positive and negative MSC markers, potential markers of enhanced chondrogenesis, and immunogenic markers of the four cell groups.

Groups		FAA Normoxia	FAA Hypoxia	MCP Normoxia	MCP Hypoxia
Positive MSC markers	CD105	94.37 ± 1.61	95.02 ± 0.83	98.07 ± 1.48	96.31 ± 0.73
	CD73	99.92 ± 0.05	99.80 ± 0.07	99.88 ± 0.12	99.92 ± 0.06
	CD90	99.99 ± 0.02	99.99 ± 0.02	99.92 ± 0.14	99.40 ± 1.03
Negative MSC markers	CD34	1.52 ± 0.55	1.32 ± 0.42	1.89 ± 1.23	0.96 ± 0.6
	CD45	0.53 ± 0.31	0.25 ± 0.25	1.00 ± 0.49	0.65 ± 0.58
Integrin markers	CD49e	99.92 ± 0.01	99.74 ± 0.27	99.96 ± 0.04	99.97 ± 0.02
	CD49b	1.78 ± 0.24	0.60 ± 0.60	19.01 ± 11.02	12.07 ± 1.73
	CD29	99.85 ± 0.04	99.65 ± 0.35	99.94 ± 0.04	99.90 ± 0.06
Potential markers of chondrogenesis	CD166	99.51 ± 0.28	99.64 ± 0.25	99.76 ± 0.24	99.85 ± 0.12
	CD146	67.92 ± 22.31	70.68 ± 22.60	89.92 ± 4.59	88.69 ± 5.98
	Podoplanin	90.66 ± 0.84	95.02 ± 4.22	76.67 ± 13.16	82.61 ± 19.5
Immunogenic markers	HLA-ABC	99.91 ± 0.15	99.80 ± 0.27	99.67 ± 0.28	99.88 ± 0.15
	HLADR	2.54 ± 1.51	3.45 ± 1.39	1.64 ± 0.69	2.37 ± 2.45
	CD80	3.27 ± 1.48	2.83 ± 2.29	3.04 ± 0.48	2.20 ± 1.26
	CD86	0.94 ± 0.35	0.54 ± 0.13	1.68 ± 0.86	0.88 ± 0.42
	CD14	1.28 ± 0.84	0.57 ± 0.69	2.05 ± 0.97	1.59 ± 1.17

When MSC markers were compared, all groups displayed a high expression of positive markers CD105, CD73, and CD90, and a low expression of negative markers CD34, CD45, and CD14. With regard to potential markers of enhanced chondrogenesis, all groups showed comparable levels of CD166 and Podoplanin, with the MCPs expressing considerably higher levels of CD146. Comparison of the integrin markers showed significantly higher levels of CD49b with MCPs, but comparable levels of CD49e and CD29 between the groups. Concerning the immunogenic markers, all groups displayed a comparable and positive expression of HLA-ABC, with a low expression of MHC II (HLA-DR) and its co-stimulatory molecules (CD80 and CD86). Data are expressed as percentage mean ± standard error mean (n = 3).

### qRT-PCR

**Figure 4:**
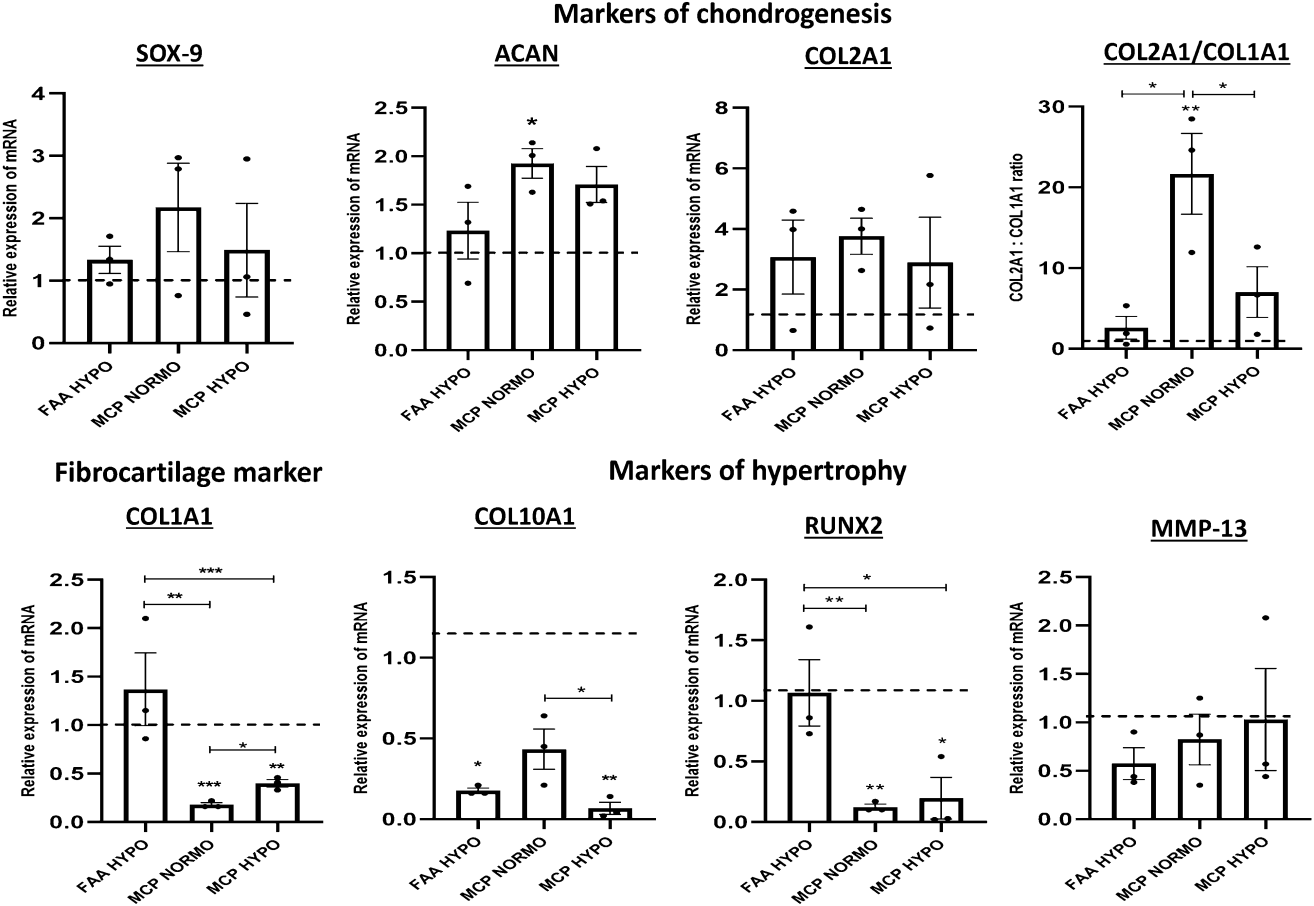
Relative mRNA expression encoding chondrogenic markers (SOX-9, ACAN, COL2A1), fibrocartilage marker (COL1A1), and hypertrophic markers (COL10A1, RUNX2, and MMP-13) were determined by qRT-PCR. All results were normalized to the housekeeping gene, GAPDH(ΔCt), and ΔΔCt was obtained in comparison to the FAA-CP Normoxia group. 2^− ΔΔCt values are expressed as mean ± standard error mean (*P < 0.05, **P < 0.01). The FAA-CP normoxia levels are represented as dotted lines. The experiments were conducted with three biological samples (n = 3) in two technical replicates. Overall, MCP-N showed considerably higher levels of chondrogenic markers with significantly higher expression of ACAN. When the functional COL2A1/COLA1 ratio was derived, MCP-Normoxia showed the highest ratio as compared to the other groups. Comparison of the fibrocartilage and hypertrophic markers showed that MCP-Normoxia expressed significantly lower levels of COL1A1 and RUNX2 as compared to FAA-CP-Normoxia and FAA-CP-Hypoxia. Both FAA-CP-Hypoxia and MCP-Hypoxia showed significantly lower levels of COL10A1 when compared to their normoxic counterparts.

With reference to markers of chondrogenesis, a high mRNA expression of ACAN and a moderate expression of SOX-9 and COL2A1 was seen in all the groups with no significant difference between them, except for ACAN (Fig. 4). Normoxia MCP displayed a stronger expression of the gene ACAN when compared to normoxia FAA-CP (*P* = 0.027). An analysis of the markers of hypertrophy showed differences between the two chondroprogenitor groups. A moderate to low expression of COL10A1, RUNX2, and MMP13 was observed in all the groups (Fig. 4). An intergroup comparison revealed that the MCPs showed remarkably lower levels of COL1A1 (fibrocartilage marker) and RUNX2 (key transcription factor for the genesis of osteoblast) when compared with FAA-CPs [(a) COL1A1: normoxia MCP Vs FAA-CPs, *P* = 0.000, hypoxia MCP vs FAA-CPs, *P* < 0.005, (b) RUNX2: normoxia MCP Vs FAA-CPs, *P* = 0.024, hypoxia MCP vs FAA-CPs, *P* = 0.009]. Only hypoxia MCPs exhibited significantly lower levels of COL10A1, another marker of osteogenesis and hypertrophy, as compared to normoxia FAA-CPs (*P* = 0.002). A comparison of normoxia FAA-CPs and MCPs to their hypoxia counterparts showed that hypoxic conditions reduced the expression of COL10A1 in both groups (*P* = 0.030 and *P* = 0.014 for FAA-CP and MCP respectively). Additionally, normoxia MCPs showed decreased levels of COL1A1 than hypoxia MCPs (*P* = 0.013). Although there was no difference seen with the expression of the mature type II collagen between the groups, the functional COL2A1/COL1A1 ratio was significantly increased in normoxia MCPs as compared to the other three groups (*P* < 0.05). Overall, we observed that normoxia MCPs showed higher levels of chondrogenic genes and lower levels of hypertrophic markers. As described in the methods section, the FAA-CPs and MCPs were cultured using standard culture media recommended and reported for these cells in previous studies.

### Multilineage differentiation, confirmatory staining, and total GAG/DNA content

**Figure 5:**
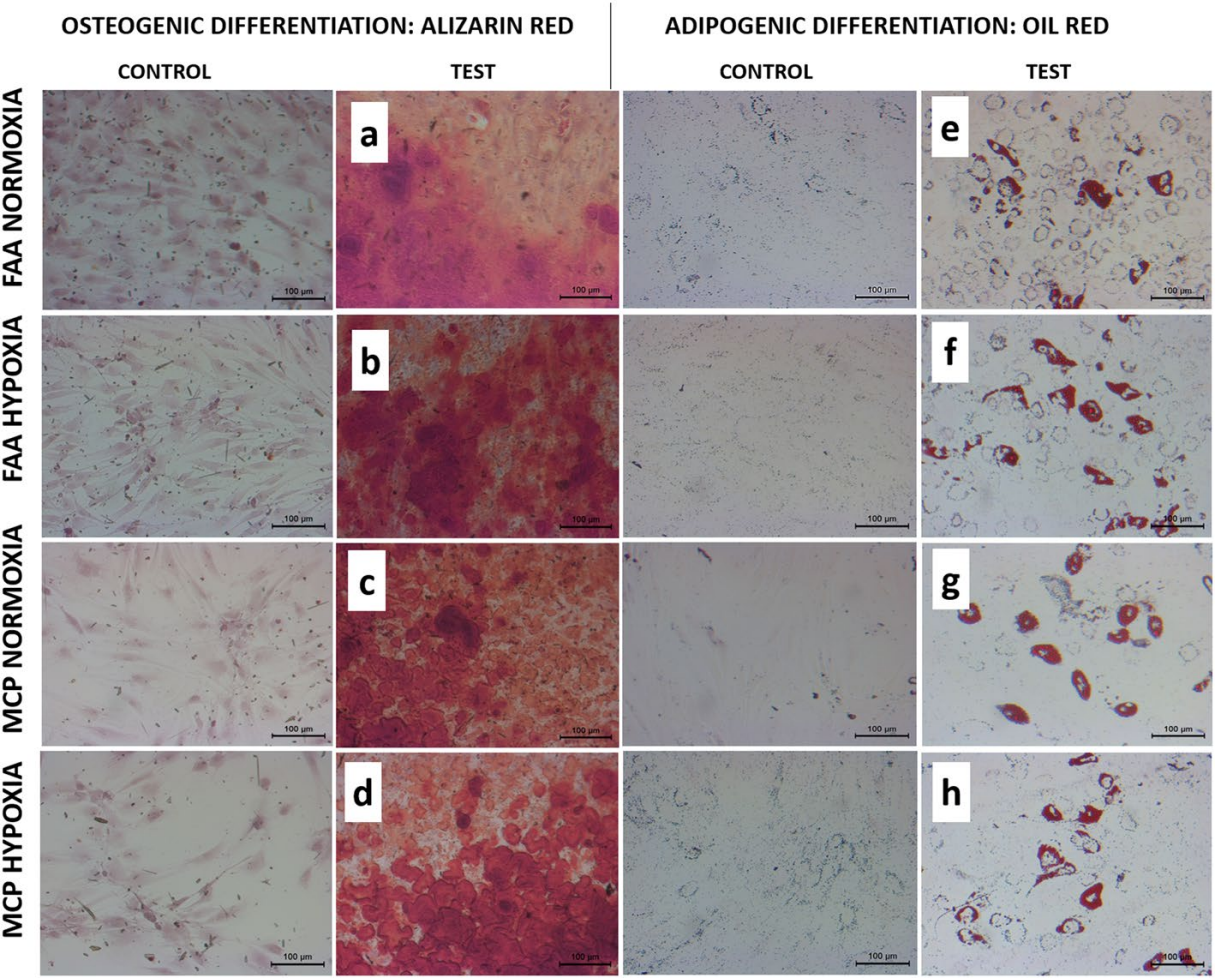
Representative microscopic images of Alizarin red (**a**–**d**) and Oil Red O (**e**–**h**) staining to confirm osteogenic and adipogenic differentiation respectively. Magnification: 20X. All groups showed comparable stain uptake confirming the presence of calcium deposition and lipid-droplet formation. Controls did not show any uptake of stain.

**Figure 6:**
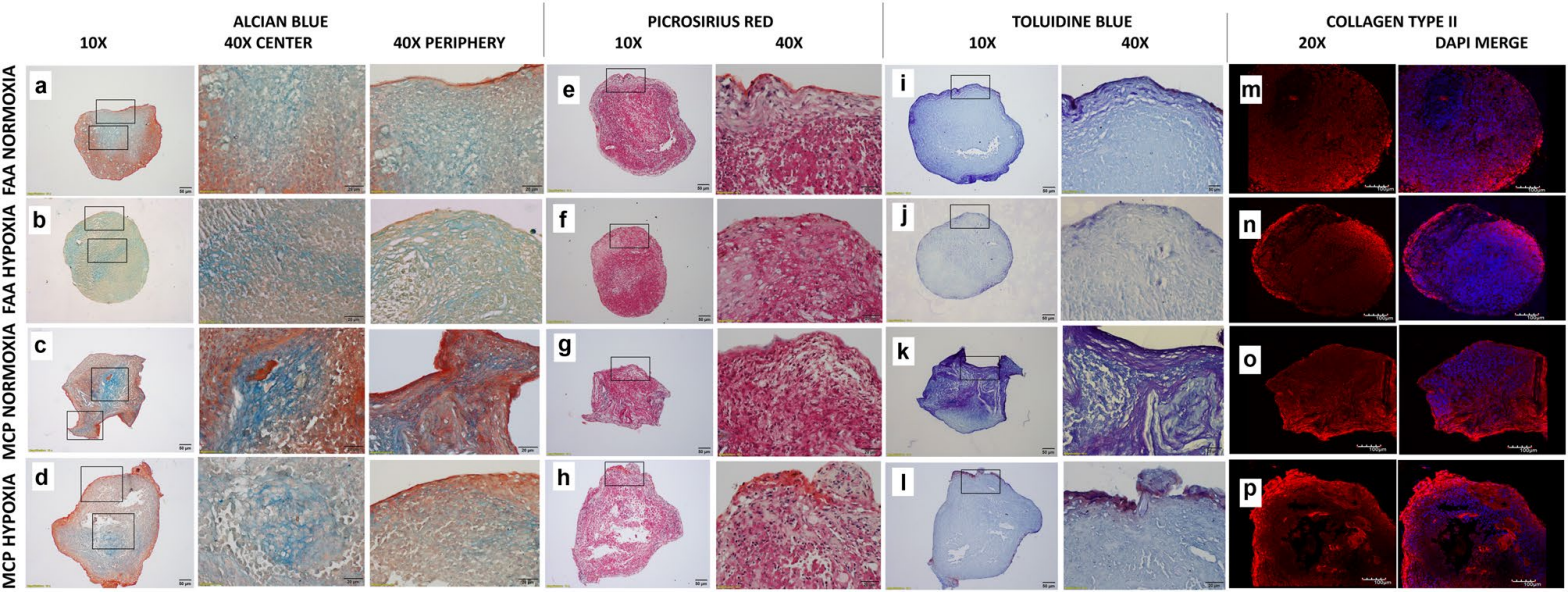
Representative image of the three independent experiments. Histological staining of chondrogenic differentiated chondroprogenitor pellets under normoxic and hypoxic culture conditions: FAA-CP-Normoxia, FAA-CP-Hypoxia, MCP-Normoxia, MCP-Hypoxia. The formed pellets (5 × 10^5^) were grown in StemPro chondrogenic medium for a period of 3 weeks. Greater uptake of Alcian Blue (**a**–**d**) and Toluidine blue (**i**–**l**) was observed with the MCP-Normoxia pellets. All groups displayed comparable Picrosirius red uptake (**e**–**h**). Immunofluorescence imaging of the pellets for collagen type II (**m**-**p**) showed similar intensity with all groups. Magnification: scale bar (50 µm or 20 µm).

**Figure 7:**
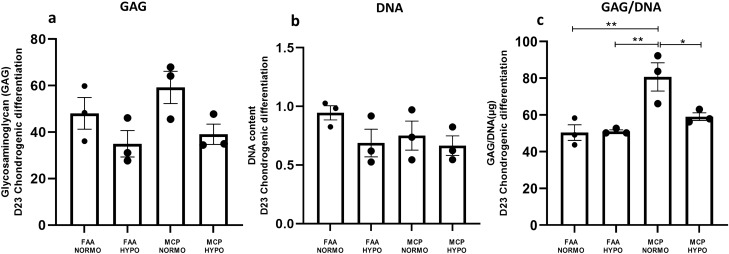
(**a**) Estimation of GAG, (**b**) DNA and (**c**) total GAG/DNA content in the chondrogenic differentiated pellet was performed following papain digestion and quantified using the dimethyl methylene blue dye method. One way-ANNOVA for GAG: 0.084, DNA: 0.25 and GAG/DNA: 0.0047. MCP-Normoxia demonstrated significantly higher levels of GAG/DNA when compared to normoxia FAA-CP (*P* = 0.001), hypoxia FAA-CP (*P* = 0.002), and hypoxia MCP (*P* = 0.01). All values are expressed as Mean ± standard error mean (*P < 0.05, **P < 0.01), from three biological samples (n = 3). GAG: glycosaminoglycan.

All the study groups demonstrated trilineage differentiation potential. When adipogenic and osteogenic potentials were qualitatively assessed using Oil Red O (lipid droplet accumulation) and Alizarin red S (calcified matrix deposition) respectively, all groups displayed positive staining (Fig. 5). Routine histological analyses of the pellets following chondrogenic differentiation showed that all the groups displayed comparable staining for Picrosirius red (Fig. 6e–h), except for normoxia MCPs, which displayed a stronger uptake for Alcian blue and Toluidine blue (Fig. 6a–d, i–l). Immunohistochemical staining for Collagen type II protein showed positive expression intensity in all the groups, with no apparent difference between them (Fig. 6m–p). When the chondrogenic induced pellets were digested and the DNA and GAG contents were measured, the normoxia MCPs demonstrated significantly higher levels of GAG/DNA as compared to the normoxia FAA-CP (*P* = 0.001), the hypoxia FAA-CP (*P* = 0.002), and the hypoxia MCP (*P* = 0.009) (Fig. 7, Supplementary Table S3). The higher levels of extracellular matrix (ECM) synthesis, observed with normoxia MCP, corroborated with the gene expression analysis for chondrogenesis.

## Discussion

The clinical goal of cartilage repair strategies is to produce a repair tissue that has identical functional and mechanical qualities as the hyaline articular cartilage. Though a multitude of reports using cell-based therapy has demonstrated therapeutic efficiency, the ensuing fibrocartilage tissue tends to be a poor substitute for long-term subsistence^39^. The discovery of cartilage resident progenitors, displaying superior chondrogenic potential and lower hypertrophic tendencies, has led to the evaluation of their use for tissue engineering applications^23^. However, clarification of their biological properties is warranted before their therapeutic use can be considered.

The main goal of this study was to compare the biological properties of resident cartilage progenitors which were derived using two distinct, but standard methods of isolation and expansion, focusing on their prospects for cartilage repair. We compared FAA-CPs and MCPs and evaluated their properties based on growth kinetics, immunophenotyping, and multilineage differentiation capacity under normoxic and hypoxic culture conditions. Further, the chondrogenic commitment was also compared, by analyzing the matrix deposition and gene expression of common chondrogenic and hypertrophic markers.

There exists a positive correlation between morphological cellular changes and chondrogenesis^40^. Spindle-shaped and fibroblast-like chondroprogenitors transform into spherical chondrocyte-like cells^41^. The changes that occur at the cellular level are the result of events that occur at the molecular level. Our results show that MCPs displayed similar morphological features and cell cycle patterns as FAA-CPs, even in the absence of additional exogenous growth factors. The isolation of chondroprogenitors in both groups required only a minimal quantity of starting cartilage material. The antigen expression pattern showed that both chondroprogenitor groups complied with the minimal ISCT criteria for MSCs with normoxia MCPs displaying significantly higher levels of CD105. CD105, in addition to being a marker for MSC, has also been reported to be a predictive marker for chondrogenesis^42–45^. With regard to the integrin markers, the MCPs displayed comparable levels of the heterodimeric fibronectin receptor (CD49e/CD29), even without treatment to the fibronectin surface. This result corroborated with a recent report that questioned the specificity of the integrin as a distinctive marker for migratory chondroprogenitors^46^. However, the expression of CD49b was higher in MCPs, similar to a recent report categorizing them to be distinctive for MCPs^47^. CD146, a known marker of enhanced chondrogenesis, was notably higher with the MCP groups, though not significant due to a single donor variance. Cell proliferation in eukaryotic cells is controlled by the precise transition from G1 to S phases of the cell cycle^48^. We observed that hypoxia groups displayed higher G1 percentages than their normoxia counterparts, however, only normoxia FAA-CPs presented the highest occurrence at the S phase. Concerning G2M phase occurrence^49^, hypoxia reduced the arrest in this phase indicating their preponderance for increased cell proliferation. These finding though not statistically significant necessitates further in-depth evaluation to understand the complex mechanisms that regulate the temporal order of transcription activation and inactivation for the distinct groups.

SOX-9 and RUNX2 are the principal regulators of chondrogenesis with different functional roles. SOX-9 plays a vital role in articular cartilage formation, while RUNX2 is primarily involved in the hypertrophic maturation of cells^50^. Both transcription factors are expressed through the entire process of chondrogenesis, starting from mesenchymal condensation and ending with terminal chondrocyte hypertrophy. Aggrecan and type II collagen protein, being the major components of the articular cartilage ECM, have also been categorized as markers of chondrocyte phenotype for in-vitro studies^51^. Thus, when considering FAA-CPs and MCPs for their distinct use in cartilage regeneration, a number of variations were observed between the two cell populations. The molecular analysis of normoxia MCPs showed greater expression of the chondrogenic markers SOX-9 and COL2A1, with a significantly higher expression with ACAN and COL2A1/COL1A1 ratio. These findings corroborate with other studies reporting higher expression of primary chondrogenic transcription factor SOX9 and stemness marker namely KLF4 in adipose-derived MSCs^52^ and increased proliferation of MSCs^53^ grown under normoxia as compared to hypoxic cultures. The RT-PCR comparison should be viewed in light of the fact that the undifferentiated cells were grown with their recommended standard media with growth factors as per their requirement, and not under a single formulation. Furthermore, an analysis of the GAG/DNA ratio obtained from chondrogenically differentiated pellets showed that normoxia MCPs outperformed the other cell groups, additionally displaying stronger uptake for glycosaminoglycans with Alcian blue and Toluidine blue.

Among the several molecular transcription factors reported to induce hypertrophy of chondrocytes leading to cell apoptosis and endo-vascularization, RUNX2 plays a major role^54,55^. Additionally, hypoxia has been reported to suppress terminal differentiation of chondrocytes with evidence of hypoxia-inducible factor-α repressing the expression of RUNX2. An evaluation of the limitation to hypertrophic commitment showed that, regardless of the microenvironment, both normoxia and hypoxia MCPs showed significantly lower levels of RUNX2 and COL1A1. Although hypoxic conditions did not induce a significant difference in the expression of chondrogenic genes, it favoured a significant decrease of COL10A1^56^, a known biomarker of terminally differentiated and hypertrophic chondrocytes, within the FAA-CP and MCP groups respectively. In accordance with our findings, a recent study demonstrated that equine MSCs isolated from synovial membrane and bone marrow showed reduced levels of COL10A1 when cultured under hypoxic conditions without any effect on chondrogenesis markers^57^. When results for trilineage differentiation were compared, a positive uptake with a similar pattern of staining was observed among all the groups, showing comparable differentiation potential.

The work reported in this study provides the first comprehensive comparison of the chondrogenic potential of human FAA-CPs and MCPs, in addition to investigating the effect of hypoxia on chondrogenesis. This study is also the first attempt to evaluate the properties of two types of progenitors derived from the same source of cartilage, and also utilizing human biological tissue. Although our results suggest that migratory chondroprogenitors cultured under normoxic conditions demonstrate higher chondrogenic potential and limited commitment to hypertrophy, hypoxia cultures display lower levels of collagen type 10, a finding that merits further evaluation to understand their additional implications for chondrogenesis. Thus, further in-depth surfaceome and proteomic analysis would help understand the beneficial effect of varying culture conditions on chondrogenesis. Since the chondroprogenitors were isolated from osteoarthritic samples, the inflammatory microenvironment could have influenced their biological properties. Further evaluation using cells obtained from non-diseased joints, using similar media formulations, and revalidation using in-vivo study models would merit further understanding of their potential therapeutic application in the field of cartilage repair.

## Supplementary Information

Supplementary Figure S1.

Supplementary Table S1.

Supplementary Table S2.

Supplementary Table S3.

## References

[1] Sophia Fox AJ, Bedi A, Rodeo SA (2009). The basic science of articular cartilage. Sports Health.

[2] Martín AR, Patel JM, Zlotnick HM, Carey JL, Mauck RL (2019). Emerging therapies for cartilage regeneration in currently excluded ‘red knee’ populations. NPJ Regen. Med..

[3] Harris JD (2011). Failures, re-operations, and complications after autologous chondrocyte implantation—A systematic review. Osteoarthr. Cartil..

[4] Pareek A (2016). Long-term outcomes after autologous chondrocyte implantation. Cartilage.

[5] Freitag, J. *et al.* Mesenchymal stem cell therapy in the treatment of osteoarthritis: reparative pathways, safety and efficacy – a review. *BMC Musculoskelet Disord* **17**, (2016). 10.1186/s12891-016-1085-9 PMC4880954 27229856

[6] Liu X (2017). High osteogenic potential of adipose- and muscle-derived mesenchymal stem cells in spinal-ossification model mice. Spine.

[7] Dowthwaite GP (2004). The surface of articular cartilage contains a progenitor cell population. J. Cell. Sci..

[8] Koelling S (2009). Migratory chondrogenic progenitor cells from repair tissue during the later stages of human osteoarthritis. Cell Stem Cell.

[9] Hayes AJ, Tudor D, Nowell MA, Caterson B, Hughes CE (2008). Chondroitin sulfate sulfation motifs as putative biomarkers for isolation of articular cartilage progenitor cells. J. Histochem. Cytochem..

[10] McCarthy HE, Bara JJ, Brakspear K, Singhrao SK, Archer CW (2012). The comparison of equine articular cartilage progenitor cells and bone marrow-derived stromal cells as potential cell sources for cartilage repair in the horse. Vet. J..

[11] Xue K (2019). Cartilage progenitor cells combined with PHBV in cartilage tissue engineering. J. Transl. Med..

[12] Vinod E, Parameswaran R, Amirtham SM, Rebekah G, Kachroo U (2021). Comparative analysis of human bone marrow mesenchymal stem cells, articular cartilage derived chondroprogenitors and chondrocytes to determine cell superiority for cartilage regeneration. Acta Histochem.

[13] Vinod E, Kachroo U, Rebekah G, Yadav BK, Ramasamy B (2020). Characterization of human articular chondrocytes and chondroprogenitors derived from non-diseased and osteoarthritic knee joints to assess superiority for cell-based therapy. Acta Histochem..

[14] Williams R (2010). Identification and clonal characterisation of a progenitor cell sub-population in normal human articular cartilage. PLoS ONE.

[15] Nelson L, McCarthy HE, Fairclough J, Williams R, Archer CW (2014). Evidence of a viable pool of stem cells within human osteoarthritic cartilage. Cartilage.

[16] Yu Y, Zheng H, Buckwalter JA, Martin JA (2014). Single cell sorting identifies progenitor cell population from full thickness bovine articular cartilage. Osteoarthr. Cartil..

[17] Su X (2015). CD146 as a new marker for an increased chondroprogenitor cell sub-population in the later stages of osteoarthritis. J. Orthop. Res..

[18] Seol D (2012). Chondrogenic progenitor cells respond to cartilage injury. Arthritis Rheum..

[19] Levato R (2017). The bio in the ink: Cartilage regeneration with bioprintable hydrogels and articular cartilage-derived progenitor cells. Acta Biomater..

[20] Kachroo U (2020). Comparison of human platelet lysate versus fetal bovine serum for expansion of human articular cartilage-derived chondroprogenitors. Cartilage.

[21] Elsaesser, A. F. *et al.* Characterization of a migrative subpopulation of adult human nasoseptal chondrocytes with progenitor cell features and their potential for in vivo cartilage regeneration strategies. *Cell Biosci* **6**, (2016). 10.1186/s13578-016-0078-6 PMC4752797 26877866

[22] Dominici M (2006). Minimal criteria for defining multipotent mesenchymal stromal cells. The International Society for Cellular Therapy position statement. Cytotherapy.

[23] Vinod E, Parameswaran R, Ramasamy B, Kachroo U (2020). Pondering the potential of hyaline cartilage-derived chondroprogenitors for tissue regeneration: A systematic review. CARTILAGE.

[24] Wang K (2019). Chondrogenic progenitor cells exhibit superiority over mesenchymal stem cells and chondrocytes in platelet-rich plasma scaffold-based cartilage regeneration. Am. J. Sports Med..

[25] Ghone NV, Grayson WL (2012). Recapitulation of mesenchymal condensation enhances in vitro chondrogenesis of human mesenchymal stem cells. J. Cell. Physiol..

[26] Handorf AM, Li W-J (2014). Induction of mesenchymal stem cell chondrogenesis through sequential administration of growth factors within specific temporal windows. J. Cell Physiol..

[27] Lee H-H (2013). Hypoxia enhances chondrogenesis and prevents terminal differentiation through PI3K/Akt/FoxO dependent anti-apoptotic effect. Sci. Rep..

[28] Fermor B (2007). Oxygen, nitric oxide and articular cartilage. Eur. Cell Mater..

[29] Meretoja VV, Dahlin RL, Wright S, Kasper FK, Mikos AG (2013). The effect of hypoxia on the chondrogenic differentiation of co-cultured articular chondrocytes and mesenchymal stem cells in scaffolds. Biomaterials.

[30] Schrobback K (2012). Effects of oxygen and culture system on in vitro propagation and redifferentiation of osteoarthritic human articular chondrocytes. Cell Tissue Res..

[31] Coyle CH, Izzo NJ, Chu CR (2009). Sustained hypoxia enhances chondrocyte matrix synthesis. J. Orthop. Res..

[32] Anderson DE, Markway BD, Weekes KJ, McCarthy HE, Johnstone B (2018). Physioxia promotes the articular chondrocyte-like phenotype in human chondroprogenitor-derived self-organized tissue. Tissue Eng. Part A.

[33] Anderson DE, Markway BD, Bond D, McCarthy HE, Johnstone B (2016). Responses to altered oxygen tension are distinct between human stem cells of high and low chondrogenic capacity. Stem Cell Res. Ther..

[34] Joos H, Wildner A, Hogrefe C, Reichel H, Brenner RE (2013). Interleukin-1 beta and tumor necrosis factor alpha inhibit migration activity of chondrogenic progenitor cells from non-fibrillated osteoarthritic cartilage. Arthritis Res. Ther..

[35] Matta C (2015). Purinergic signalling is required for calcium oscillations in migratory chondrogenic progenitor cells. Pflugers Arch..

[36] Swart GWM (2002). Activated leukocyte cell adhesion molecule (CD166/ALCAM): Developmental and mechanistic aspects of cell clustering and cell migration. Eur. J. Cell Biol..

[37] Dicks, A. *et al.* Prospective isolation of chondroprogenitors from human iPSCs based on cell surface markers identified using a CRISPR-Cas9-generated reporter. *bioRxiv* 675983 (2019) 10.1101/675983. 10.1186/s13287-020-01597-8 PMC7026983 32070421

[38] Chan CKF (2018). Identification of the human skeletal stem cell. Cell.

[39] Armiento AR, Alini M, Stoddart MJ (2019). Articular fibrocartilage—why does hyaline cartilage fail to repair?. Adv. Drug Deliv. Rev..

[40] Augustyniak E, Trzeciak T, Richter M, Kaczmarczyk J, Suchorska W (2015). The role of growth factors in stem cell-directed chondrogenesis: a real hope for damaged cartilage regeneration. Int. Orthop. (SICOT).

[41] Matta C, Mobasheri A (2014). Regulation of chondrogenesis by protein kinase C: Emerging new roles in calcium signalling. Cell Signal.

[42] Bernstein P, Sperling I, Corbeil D, Hempel U, Fickert S (2013). Progenitor cells from cartilage–no osteoarthritis-grade-specific differences in stem cell marker expression. Biotechnol. Prog..

[43] Fan W (2016). CD105 promotes chondrogenesis of synovium-derived mesenchymal stem cells through Smad2 signaling. Biochem. Biophys. Res. Commun..

[44] Ozbey O, Sahin Z, Acar N, Ustunel I (2010). Distribution of CD105 and CD166 positive cells in the proximal epiphysis of developing rat humerus. Histol. Histopathol..

[45] Wang M (2013). Experimental study on CD105+/CD166+ cells and its chondrogenic potential in early osteoarthritis cartilage. Zhongguo Xiu Fu Chong Jian Wai Ke Za Zhi.

[46] Benz K, Stippich C, Freudigmann C, Mollenhauer JA, Aicher WK (2013). Maintenance of ‘stem cell’ features of cartilage cell sub-populations during in vitro propagation. J. Transl. Med..

[47] Matta C (2019). Molecular phenotyping of the surfaceome of migratory chondroprogenitors and mesenchymal stem cells using biotinylation, glycocapture and quantitative LC-MS/MS proteomic analysis. Sci. Rep..

[48] Bertoli C, Skotheim JM, de Bruin RAM (2013). Control of cell cycle transcription during G1 and S phases. Nat. Rev. Mol. Cell. Biol..

[49] Stark GR, Taylor WR (2004). Analyzing the G2/M checkpoint. Methods Mol. Biol..

[50] Goldring MB (2012). Chondrogenesis, chondrocyte differentiation, and articular cartilage metabolism in health and osteoarthritis. Ther. Adv. Musculoskelet. Dis..

[51] Frazer A, Bunning RA, Thavarajah M, Seid JM, Russell RG (1994). Studies on type II collagen and aggrecan production in human articular chondrocytes in vitro and effects of transforming growth factor-beta and interleukin-1beta. Osteoarthr. Cartil..

[52] Zhao AG, Shah K, Freitag J, Cromer B, Sumer H (2020). Differentiation potential of early- and late-passage adipose-derived mesenchymal stem cells cultured under hypoxia and normoxia. Stem Cells Int..

[53] Holzwarth C (2010). Low physiologic oxygen tensions reduce proliferation and differentiation of human multipotent mesenchymal stromal cells. BMC Cell Biol..

[54] Eames BF, Sharpe PT, Helms JA (2004). Hierarchy revealed in the specification of three skeletal fates by Sox9 and Runx2. Dev. Biol..

[55] Wang GL, Jiang BH, Rue EA, Semenza GL (1995). Hypoxia-inducible factor 1 is a basic-helix-loop-helix-PAS heterodimer regulated by cellular O2 tension. Proc. Natl. Acad. Sci. U. S. A..

[56] Shen G (2005). The role of type X collagen in facilitating and regulating endochondral ossification of articular cartilage. Orthod. Craniofac. Res..

[57] Gale AL, Mammone RM, Dodson ME, Linardi RL, Ortved KF (2019). The effect of hypoxia on chondrogenesis of equine synovial membrane-derived and bone marrow-derived mesenchymal stem cells. BMC Vet. Res..

[58] Teixeira FG (2015). Do hypoxia/normoxia culturing conditions change the neuroregulatory profile of Wharton Jelly mesenchymal stem cell secretome?. Stem Cell Res. Ther..

