# Supplementary figures and images for: Migratory chondroprogenitors retain superior intrinsic chondrogenic potential for regenerative cartilage repair as compared to human fibronectin derived chondroprogenitors
Source: Sci Rep. 2021 Dec 8;11:23685. doi: 10.1038/s41598-021-03082-5 (PMC8654938; doi:10.1038/s41598-021-03082-5)

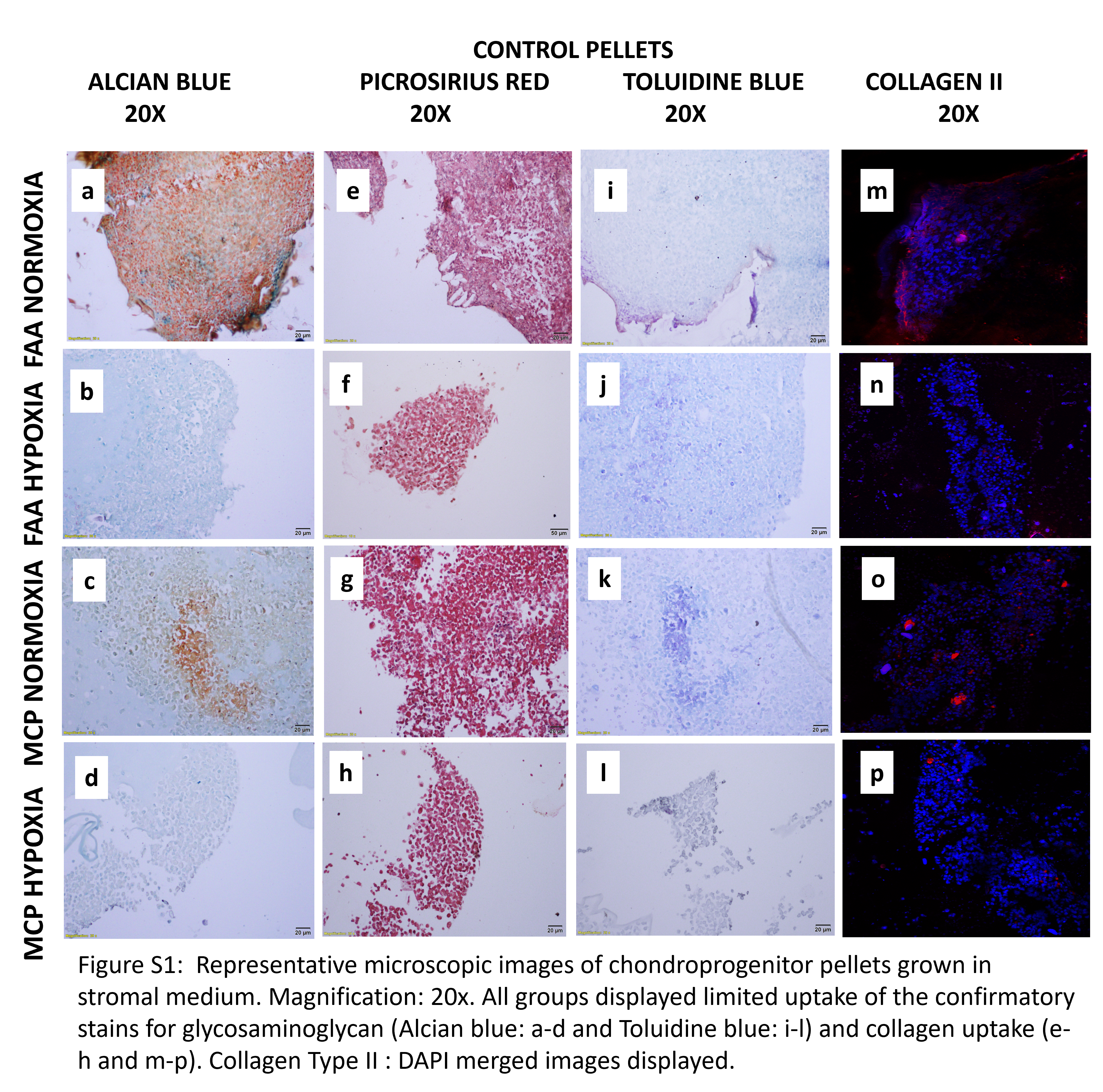

Supplement: Supplementary file 1 — Supplementary Figure S1. [file 41598_2021_3082_MOESM1_ESM.tif]
