# Supplementary material for: Migratory chondroprogenitors retain superior intrinsic chondrogenic potential for regenerative cartilage repair as compared to human fibronectin derived chondroprogenitors
Source: Sci Rep. 2021 Dec 8;11:23685. doi: 10.1038/s41598-021-03082-5 (PMC8654938; doi:10.1038/s41598-021-03082-5)
Supplement: Supplementary file 2 — Supplementary Table S1. [file 41598_2021_3082_MOESM2_ESM.pdf]

Supplementary Table S1: List of antibodies used for characterization of chondroprogenitors by flow cytometric analysis. MSC: mesenchymal stem cell, CD: cluster of differentiation, FITC: fluorescein isothiocyanate, PE: phycoerythrin, APC: allophycocyanin, BB515: Horizon brilliant blue 515, BV421: Brilliant violet 421 and V500: Violet 500.

<table>
<tr><th>Groups</th><th colspan="2">Surface Markers</th><th>Fluorochrome Conjugate</th><th>Catalogue number</th><th>Source</th></tr>
<tr><td rowspan="3">Group I:
Positive MSC markers</td><td colspan="2">CD105: Endoglin glycoprotein</td><td>FITC</td><td>561443</td><td>BD Bioscience</td></tr>
<tr><td colspan="2">CD73: Ecto-5’- nucleotidase</td><td>PE</td><td>550257</td><td>BD Bioscience</td></tr>
<tr><td colspan="2">CD90: Thymus cell antigen 1</td><td>PE</td><td>555596</td><td>BD Bioscience</td></tr>
<tr><td rowspan="2">Group II:
Negative MSC markers</td><td>CD34</td><td rowspan="2">Hematopoietic stem cell markers</td><td>APC</td><td>560940</td><td>BD Bioscience</td></tr>
<tr><td>CD45</td><td>FITC</td><td>555482</td><td>BD Bioscience</td></tr>
<tr><td rowspan="2">Group III:
Integrin markers</td><td colspan="2">CD29: Integrin beta-1 (Iβ1)</td><td>APC</td><td>559883</td><td>BD Bioscience</td></tr>
<tr><td colspan="2">CD49e: Integrin alpha 5; (Iα5) Fibronectin receptor</td><td>PE</td><td>555617</td><td>BD Bioscience</td></tr>
<tr><td></td><td colspan="2">CD49b: Integrin alpha 2; (Iα2)</td><td>FITC</td><td>MACS 130/100337</td><td>Miltenyl Biotec</td></tr>
<tr><td>Group IV:</td><td colspan="2">CD146: Melanoma cell adhesion molecule</td><td>PE</td><td>550315</td><td>BD Bioscience</td></tr>
</table>

Potential markers of enhanced chondrogenesis	CD166: Activated leucocyte adhesion molecule	BB515	564561	BD Bioscience
	Podoplanin: Type I integral membrane glycoprotein	BV421	566456	BD Bioscience
**Group IV:** Immunogenic markers	HLA-ABC: Human Leukocyte Class I	PE	560964	BD Bioscience
	HLA-DR: Human Leucocyte Class II	V500	561225	BD Bioscience
	CD80: HLA-II costimulatory marker	BB515	565009	BD Bioscience
	CD86: HLA-II costimulatory marker	BV421	562433	BD Bioscience
	CD14: Monocyte/macrophage marker	FITC	555397	BD Bioscience
