# Supplementary material for: Migratory chondroprogenitors retain superior intrinsic chondrogenic potential for regenerative cartilage repair as compared to human fibronectin derived chondroprogenitors
Source: Sci Rep. 2021 Dec 8;11:23685. doi: 10.1038/s41598-021-03082-5 (PMC8654938; doi:10.1038/s41598-021-03082-5)
Supplement: Supplementary file 3 — Supplementary Table S2. [file 41598_2021_3082_MOESM3_ESM.docx]

Supplementary Table S2: Sequence of the primers used for RT-PCR. SOX9: sex determining region Y-box 9, ACAN: Aggrecan, COL2A1: Collagen type 2 alpha 1 chain, COL1A1: Collagen type 1 alpha 1 chain, COL10A1: Collagen type 10 alpha 1 chain, RUNX2: Runt related transcription factor-2 and MMP13: Matrix metalloproteinase type 13. GAPDH: glyceraldehyde 3-phosphate dehydrogenase

| **Gene of Interest** | **Primers (5’-3’)** | | **Accession number (reference link)** | **Product size**  **(bp)** |
| --- | --- | --- | --- | --- |
|  | **Forward primer** | **Reverse primer** |  |  |
| **SOX-9** | GACTTCCGCGACGTGGAC | GTTGGGCGGCAGGTACTG | NM_000346.4  <https://www.ncbi.nlm.nih.gov/nucleotide/1519242934> | 99 |
| **ACAN, transcript variant 1** | TCGAGGACAGCGAGGCC | TCGAGGGTGTAGCGTGTAGAGA | NM_001135.4  <https://www.ncbi.nlm.nih.gov/nucleotide/1890265422> | 85 |
| **COL2A1, transcript variant 2** | CCTGAGTGGAAGAGTGGAGAC | TTGCTGCTCCACCAGTTCTT | NM_033150.3  <https://www.ncbi.nlm.nih.gov/nucleotide/1674985896> | 149 |
| **COL1A1** | TCTGCGACAACGGCAAGGTG | GACGCCGGTGGTTTCTTGGT | NM_000088.4  <https://www.ncbi.nlm.nih.gov/nucleotide/1777425449> | 146 |
| **COL10A1** | CAAGGCACCATCTCCAGGAA | AAAGGGTATTTGTGGCAGCATATT | NM_000493.4  <https://www.ncbi.nlm.nih.gov/nucleotide/1519245829> | 70 |
| **RUNX2,**  **transcript variant 2** | CCTAAATCACTGAGGCGGTC | CAGTAGATGGACCTCGGGAA | NM_001015051.4  <https://www.ncbi.nlm.nih.gov/nucleotide/1890358904> | 91 |
| **MMP-13** | TCTGAACTGGGTCTTCCAAAA | GCATCTACTTTATCACCAATTCCT | NM_002427.4  <https://www.ncbi.nlm.nih.gov/nucleotide/1519312163> | 188 |
| **GAPDH**  **transcript variant 7** | TCAGCAATGCCTCCTGCAC | TCTGGGTGGCAGTGATGGC | NM_001357943.2  <https://www.ncbi.nlm.nih.gov/nucleotide/1676440496> | 117 |
