# Supplementary material for: Migratory chondroprogenitors retain superior intrinsic chondrogenic potential for regenerative cartilage repair as compared to human fibronectin derived chondroprogenitors
Source: Sci Rep. 2021 Dec 8;11:23685. doi: 10.1038/s41598-021-03082-5 (PMC8654938; doi:10.1038/s41598-021-03082-5)
Supplement: Supplementary file 4 — Supplementary Table S3. [file 41598_2021_3082_MOESM4_ESM.docx]

Supplementary table 3: Total glycosaminoglycan (GAG), DNA and GAG)/DNA(µg) obtained from papain digested chondrogenically differentiated chondroprogenitor pellets on day 23 following chondrogenic differentiation. Data shown includes the individual values obtained from the three biological samples (n=3). Normoxia MCP showed significantly higher levels of GAG/DNA when compared to all the groups. FAA CP: fibronectin adhesion assay derived chondroprogenitors, MCP: migratory chondroprogenitors.

|  |  | FAA CP NORMOXIA | FAA CP HYPOXIA | MCP NORMOXIA | MCP  HYPOXIA |
| --- | --- | --- | --- | --- | --- |
| GAG | Donor 1 | 59.8 | 46.1 | 68.0 | 47.7 |
|  | Donor 2 | 48.3 | 31.1 | 45.5 | 35.0 |
|  | Donor 3 | 36.1 | 27.7 | 64.2 | 34.4 |
| DNA | Donor 1 | 1.0 | 0.9 | 0.7 | 0.8 |
|  | Donor 2 | 1.0 | 0.6 | 0.5 | 0.6 |
|  | Donor 3 | 0.8 | 0.5 | 1.0 | 0.5 |
| GAG/DNA | Donor 1 | 58.3 | 50.2 | 92.2 | 57.9 |
|  | Donor 2 | 49.1 | 50.2 | 83.7 | 56.2 |
|  | Donor 3 | 43.7 | 52.7 | 66.1 | 63.0 |
